# Hybrid Endovascular and Surgical Treatment of a Traumatic Scalp Arteriovenous Fistula

**DOI:** 10.7759/cureus.49450

**Published:** 2023-11-26

**Authors:** Muzhda Subhan, Salman Shah, Saurabh Patel, Anantha Ramanathan

**Affiliations:** 1 Department of Radiology, Nassau University Medical Center, East Meadow, USA; 2 Department of Surgery, Nassau University Medical Center, East Meadow, USA

**Keywords:** angiography, vascular surgery, embolization, scalp trauma, scalp arteriovenous fistula

## Abstract

Traumatic scalp arteriovenous fistula (AVF) is a relatively rare complication of scalp trauma. Patients most commonly present with a growing pulsatile head mass. History of trauma, clinical presentation, and diagnostic imaging, including digital subtraction angiography, aid in establishing the diagnosis. Endovascular embolization is the preferred treatment modality which may be combined with surgical excision for larger complex lesions. In this case, we report the clinical and radiological features of a traumatic scalp AVF in a middle-aged man with a remote history of trauma that was treated with a two-step hybrid approach combining transarterial embolization with surgical resection. We also present a brief overview of the various treatment modalities currently employed to treat scalp AVFs.

## Introduction

Traumatic scalp arteriovenous fistula (AVF) represents a direct communication between the arterial and venous systems [1]. The inciting trauma can be recent or remote, blunt or penetrating [2]. Most scalp AVFs present with bothersome symptoms, including headaches and tinnitus in addition to a growing pulsatile mass as was in our case [3]. Traumatic AVF can range from a single communication between an artery and a vein to a complex plexiform nidus with multiple fistulous connections [4]. Treatment is necessary to not only alleviate the symptoms but also prevent serious complications like massive bleeding. Various treatment modalities are available, and the choice of treatment primarily depends on the angioarchitecture of the AVF in addition to other factors [5].

## Case presentation

History and physical exam

A 46-year-old man was referred to the surgery clinic with an ongoing issue of a painless, pulsatile swelling on the right side of his scalp. About eight years ago, he experienced a traumatic laceration to his right parietal scalp that necessitated suturing. Three years later, he noticed a pulsatile bump forming under the scar. Initially small, the mass had gradually increased in size, causing him daily headaches, dizziness while lying down, and pulsatile tinnitus on the right side.

**Figure 1:**
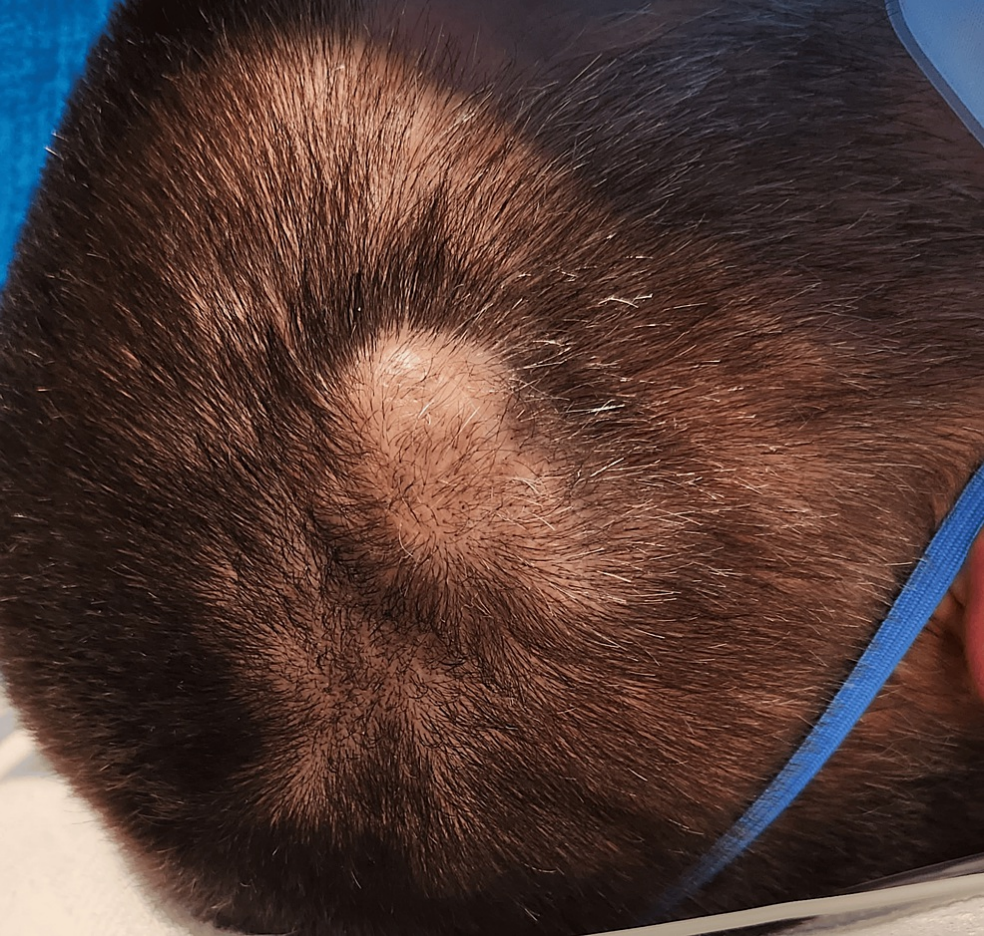
Clinical photograph showing right parietal scalp swelling.

On physical examination, a non-tender, pulsatile, fluctuant mass measuring 2x3 cm was observed on the right parietal scalp (Figure 1). Its pulsations were synchronous with the heartbeat. No erythema, skin changes or discharge was noted. The patient’s vital signs were normal.

Investigations

**Figure 2:**
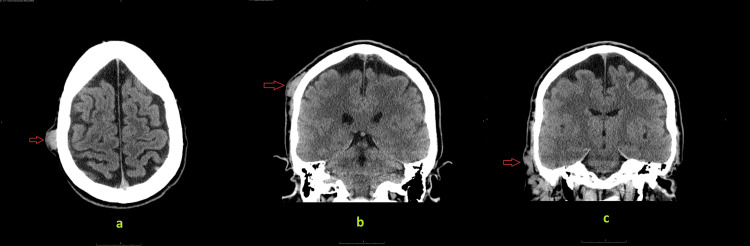
Axial and coronal contrast-enhanced CT head. Images a and b show a tangle of dilated blood vessels (arrows) in the right parietal scalp. Image c shows the tortuous and abnormally dilated vessels in the right temporal region (arrow).

**Figure 3:**
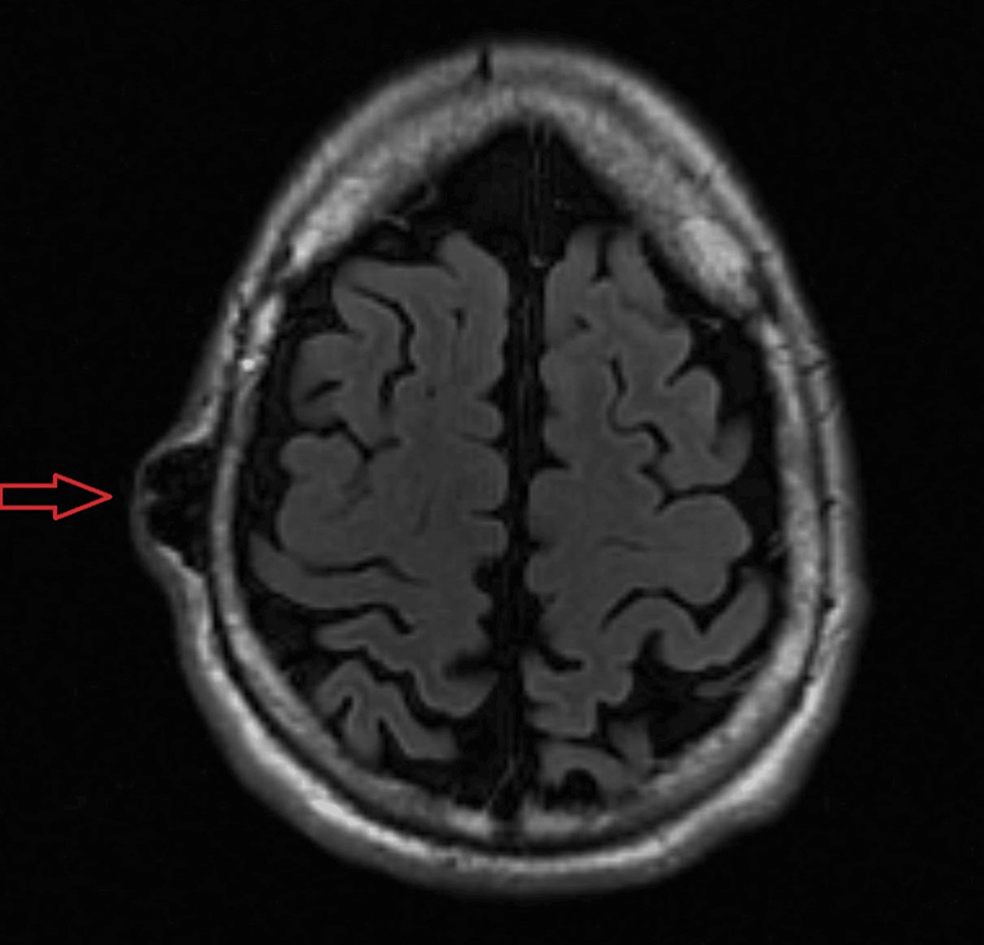
Axial T2 weighted magnetic resonance imaging (MRI) image shows a tangle of flow voids in the right parietal scalp (arrow) indicating high-flow vessels.

**Figure 4:**
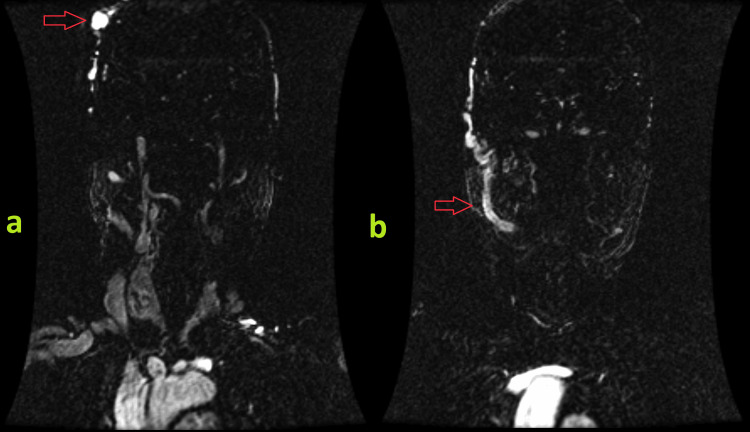
Coronal magnetic resonance angiography (MRA) images showing a tangle of vessels in the right parietal scalp (arrow in image a) and a dilated right superficial temporal vein draining the abnormal vasculature (arrow in image b).

**Figure 5:**
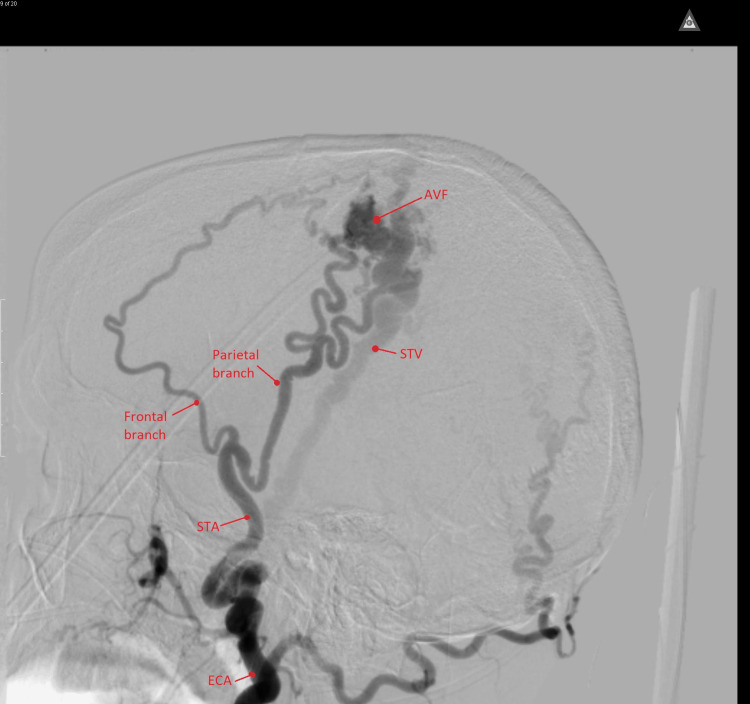
Angiogram of the right external carotid artery shows a dilated external carotid artery (ECA), superficial temporal artery (STA) and its frontal and temporal branches (arrows) feeding the plexiform arteriovenous fistula (AVF). The early filling dilated superficial temporal vein (STV) is also observed.

The patient underwent contrast-enhanced computed tomography (CT) of the head, which revealed dilated and tortuous blood vessels in the right parietal scalp, forming a nidus measuring approximately 1.9x1.1x1.7 cm. Adjacent dilated extracranial vessels were noted but no intracranial or extracranial aneurysmal dilatation or intracranial communication was found (Figure 2). A diagnosis of traumatic arteriovenous fistula was suggested, and magnetic resonance imaging (MRI) and magnetic resonance angiography (MRA) were recommended. MRI and MRA redemonstrated the mass-like tangle of vessels in the right parietal scalp along with abnormally dilated and twisted vessels extending from the vertex to the right temporal fossa. A significantly enlarged right external carotid artery and an early enhancing dilated superficial temporal vein draining into the right external jugular vein were identified (Figures 3, 4). These findings were consistent with a plexiform scalp AVF. The patient then underwent right carotid angiography which confirmed the AVF to be between the right superficial temporal artery (STA) and the right superficial temporal vein (STV). Two feeding branches of the right STA were identified: the frontal branch and the temporal branch (Figure 5). Notably, no communication was detected with the intracranial vessels. Collectively, the imaging results and clinical symptoms established the diagnosis of a post-traumatic plexiform AVF of the scalp.

Differential diagnosis

Based on the patient’s presentation of a pulsatile post-traumatic scalp swelling, three preliminary diagnoses were considered: traumatic pseudoaneurysm, traumatic AVF, and congenital arteriovenous malformation.

The CT and MRI findings were both suggestive of AVF rather than a pseudoaneurysm as no focal dilation or outpouching of an artery was observed. Instead, a tangle of dilated vessels along with an early contrast filling adjacent vein was observed, which was redemonstrated on digital subtraction angiography. These findings could either indicate a post-traumatic AVF or a congenital AVM. We established the diagnosis of a post-traumatic AVF based on the patient’s statement that he did not have the swelling prior to the trauma or for a considerable time afterward. He stated that he often checked his scar and never noticed a lump prior to the development of the swelling.

Treatment

After analyzing the angioarchitecture of the AVF, we opted to perform endovascular coil embolization via a transarterial approach. Access was obtained through the brachial artery and a microcatheter system (Truselect Microcatheter; Boston Scientific, Marlborough, MA, USA) was navigated through the proximal tortuous segment of the right STA into the anterior (frontal) arterial feeder. Embolization was performed using three microcoils (Prestige Plus Microcoil; Balt, Irvine, CA, USA) of variable sizes. The microcatheter system was then engaged into the posterior (temporal) arterial feeder and coil embolization was performed with two microcoils.

**Figure 6:**
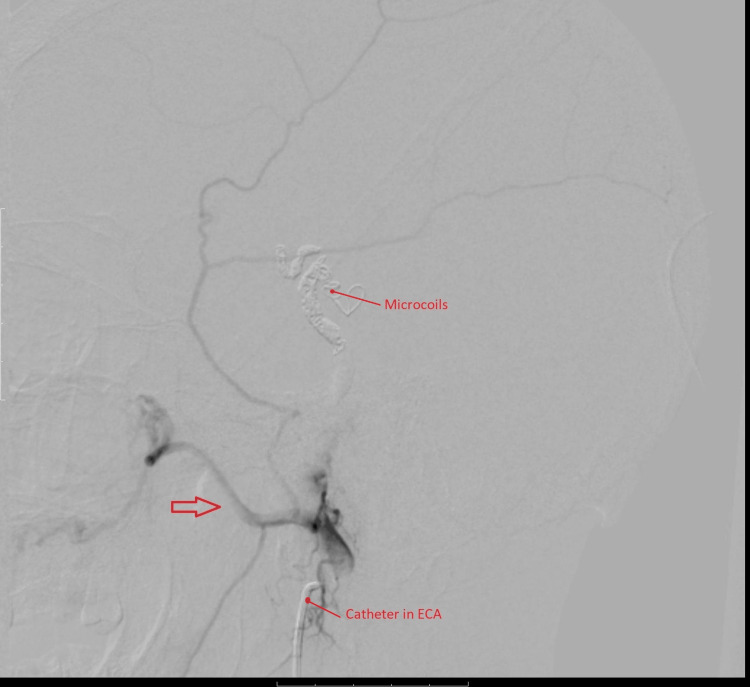
Post-embolization angiogram of the right external carotid artery shows coils in the arterial feeders and cessation of blood flow to the arteriovenous fistula (AVF). Contrast filling maxillary artery (arrow) is noted but no additional arterial feeders are observed.

Post-embolization angiogram was performed through the microcatheter which demonstrated complete occlusion of the supplying arteries (Figure 6). The microcatheter was then retracted to the proximal right external carotid artery and an additional angiogram was performed. No additional arterial feeders were observed supplying the AVF. 

Unexpectedly, during the follow-up visit two weeks later, the mass remained unchanged and pulsatile. The only improvement observed was that the patient no longer felt dizziness while lying down. On examination, a pulsation was felt posterior to the mass, indicating an additional arterial feeder, presumably from the right occipital artery. At this point surgical excision of the AVF was advised to which the patient consented.

**Figure 7:**
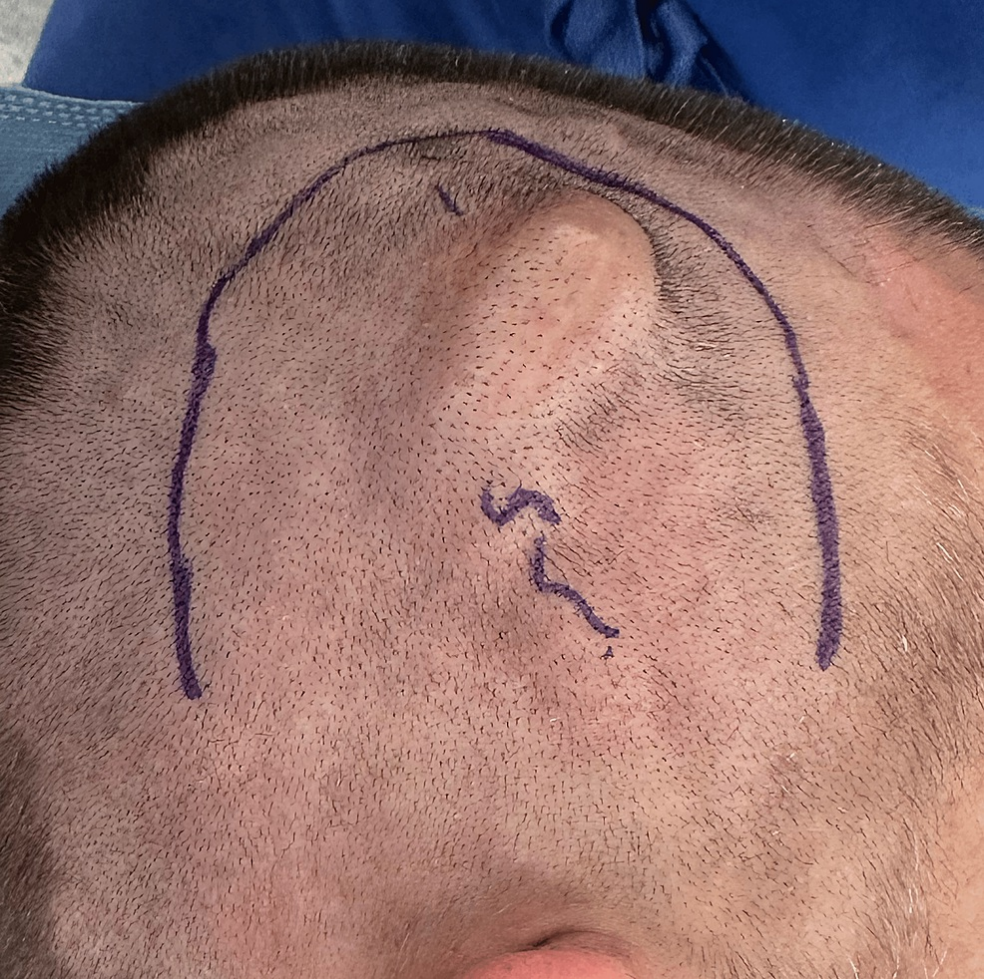
Clinical presurgical photograph showing markings for the horseshoe incision around the arteriovenous fistula (AVF). The AVF had not significantly changed from the initial presentation.

**Figure 8:**
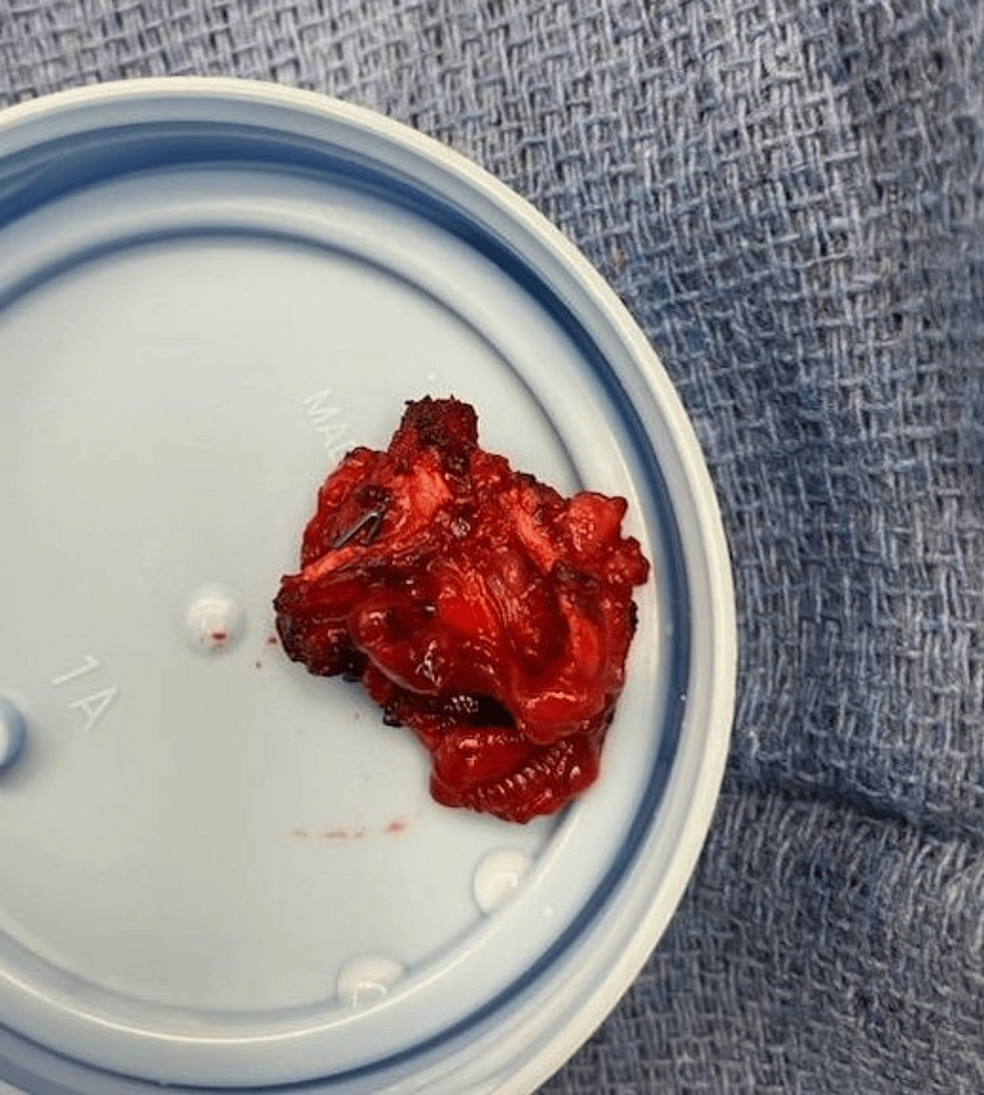
Excised plexiform arteriovenous fistula (AVF).

Under general anesthesia, a horseshoe incision was made in the right temporal region and dissected down to the level of the skull (Figure 7). Using blunt dissection, the total extent of the AVF was exposed. At least three arterial feeders were identified, one arising from the occipital artery and two from the superficial temporal artery. Each was ligated using clips or 2-0 silk, following which the AVF was completely excised (Figure 8). Hemostasis was achieved using electrocautery and bipolar coagulation forceps. The surgical wound was copiously irrigated, and the wound closed in a multilayered fashion. Lastly, a 7 Fr active drain was inserted and a dressing applied. The drain was removed the next day and the patient was recommended to follow up in the clinic after two weeks.

**Figure 9:**
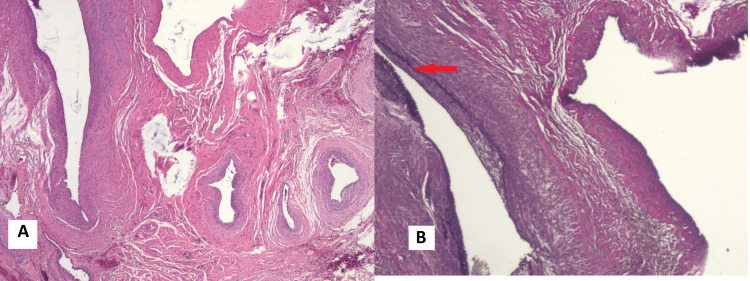
Image A shows a haematoxylin and eosin-stained microscopic section demonstrating dilated thick-walled vessels separated by connective tissue. Image B shows an elastica-stained section demonstrating an artery with internal elastic lamina (arrow) and a vein without internal elastic lamina.

Microscopic sections of the surgical sample stained with hematoxylin and eosin showed a network of dilated vascular channels separated by soft tissue. Elastica-stained samples demonstrated internal elastic lamina in some of the thick-walled vessels, consistent with arteries, while others were devoid of the internal elastic lamina indicating their venous nature (Figure 9).

Outcome and follow-up

**Figure 10:**
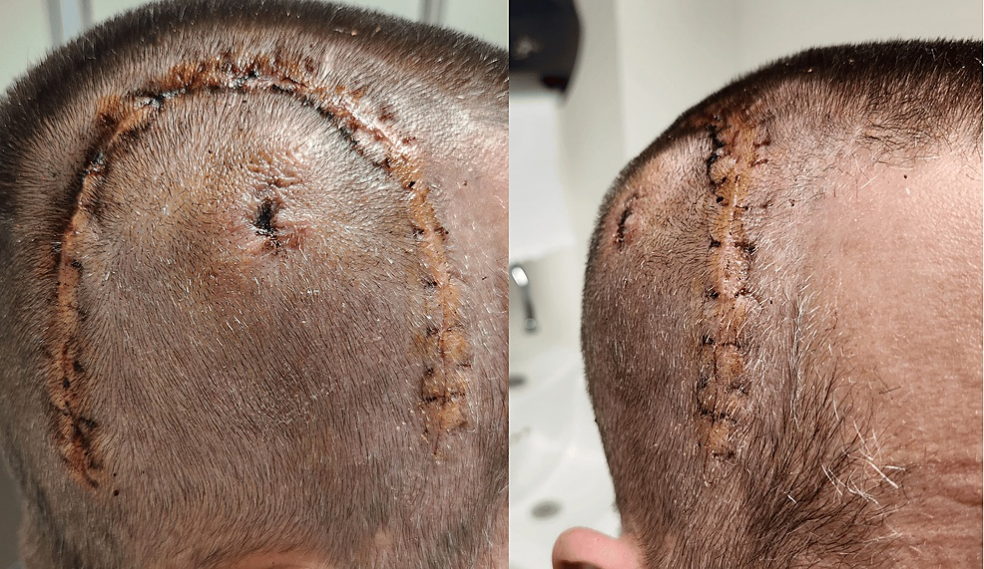
Clinical photographs (lateral and anterior views) two weeks after the surgery showing well healing incision and no residual scalp mass. The small central hole represents the site of the drain.

Patient followed up at the vascular surgery clinic as scheduled. His scar was found to be healing well, and the patient had no complaints other than about the mild swelling of the scalp (Figure 10). The sutures were removed during the visit and the patient was advised to return to full daily and work activities. He followed up again a month later with no signs of AVF recurrence.

## Discussion

Arteriovenous fistula refers to a direct connection between the arterial and venous systems without an intervening capillary bed [1]. AVFs of the scalp can occur congenitally, as a result of trauma, due to iatrogenic causes or spontaneously [3]. Traumatic AVFs of the scalp are rare with less than 50 cases reported in the literature [2]. Two pathophysiological mechanisms have been proposed for their formation: simultaneous laceration of an artery and an adjacent vein creating a single fistula between the vessels, and traumatic rupture of arterial vasa vasorum, causing proliferation of endothelial cells and formation of numerous vascular channels between the affected artery/ies and vein/s [6].

In most cases, traumatic scalp AVFs are supplied by branches of the external carotid artery which undergo hypertrophy over time. The superficial temporal and occipital arteries are the most commonly involved arteries [2]. Rare case reports have documented communication with the internal carotid system, such as the case reported by Zheng et al., where a traumatic scalp AVF was supplied by both the superficial temporal artery and branches of the internal carotid artery [7].

Based on the number of fistulas and arterial feeders, scalp AVFs are classified into three categories, summarized in Table 1 [4].

**Table 1:** Types of traumatic arteriovenous fistula (AVF).

Category	Characteristics
Type A	Single arterial feeder supplying a single fistula
Type B	Multiple arterial feeders supplying a single fistula
Type C	Multiple arterial feeders forming a plexiform with multiple fistulas and a single draining vein

The presentation of traumatic scalp AVF can occur within a few weeks or can be delayed for several years after trauma [2]. The most frequently observed symptoms include a pulsatile and gradually enlarging head mass and pulsatile tinnitus [3]. Headaches, dizziness, and fatigue may also occur, albeit less commonly. Physical examination typically reveals a soft, compressible swelling that produces a palpable thrill and bruit. Manual compression of the feeding vessels often leads to a reduction in the size of the swelling [6].

Various diagnostic imaging modalities are used to evaluate traumatic scalp AVFs [5], including ultrasound with Doppler, computed tomography angiography (CTA), MRI, MRA, and digital subtraction angiography (DSA). Diagnosis is established if the angiography (CTA, MRA, or DSA) reveals early contrast filling in a vein adjacent to the lesion during the arterial phase [8]. DSA is considered the gold standard for establishing the diagnosis as it enables visualization of the feeding arteries, draining veins, and the presence of any intracranial communication.

Therapeutic intervention is usually pursued to alleviate symptoms, prevent complications such as hemorrhage or in cases of intracranial communication, diversion of blood flow from the internal carotid system, and for cosmetic purposes. Several therapeutic modalities have been employed and are outlined in Table 2 [5].

**Table 2:** Various treatment modalities for traumatic scalp arteriovenous fistula (AVF).

Surgical treatment	Surgical excision with feeder ligation
Endovascular treatment	Transarterial embolization Transvenous embolization Direct puncture sclerotherapy or embolization
Combined treatment	Endovascular embolization followed by surgical excision

Surgical excision with feeder ligation has the lowest recurrence rate, however, if prior embolization of the arterial feeders is not performed, there is a high risk of intraoperative bleeding [3]. Other drawbacks include risk of injury to the facial nerve, incomplete removal of the AVF, and the need for scalp reconstructive surgeries in the case of larger lesions [9].

Transarterial embolization using microcoils or microvascular plugs can be therapeutic in cases of a single AVF with a single arterial feeder. However, in situations with multiple fistulous connections, transarterial embolization alone may fail to identify and embolize all the feeding arteries, as demonstrated in our case [4]. Complications such as distal migration of the coil can occur if it is undersized for the feeding artery. Therefore, transarterial embolization is preferably used as an adjunct to surgery to reduce flow to the AVF, rather than as a curative approach [10]. Transvenous embolization utilizing liquid embolic agents like Onyx has been widely and successfully employed in cases where only one draining vein is involved, avoiding the challenge of missing unidentified arterial feeders [4]. However, these agents are more difficult to control, and there is a risk of distal extension of the embolic agent, which can be hazardous if an unidentified intracranial communication of the scalp AVF is present [5]. To avoid these risks, modifications like using coils in addition to liquid embolic agents are utilized [4].

Direct puncture embolization using sclerosing agents such as n-butyl cyanoacrylate (NBCA) has also been reported, offering the advantage of bypassing the arterial feeders. This approach results in a faster procedure with reduced radiation and contrast exposure [11]. However, a dilated venous pouch and complete control of inflow and outflow are required to ensure effective occlusion and prevent distal migration of the embolic agents into the pulmonary circulation [5,12]. Additionally, larger lesions requiring a substantial amount of the sclerosing agent may carry a risk of overlying skin necrosis [13].

The choice of treatment depends on various factors, such as the size of the AVF, flow characteristics, size and number of feeding and draining vessels, availability of embolizing agents, and patient preferences. Generally, endovascular treatment is preferred [14] and can be performed alone or combined with subsequent surgical excision for residual or more complex type C lesions [4]. In our case, employed the transarterial instead of the preferred transvenous approach due to lack of liquid embolic agents at our facility. We then proceeded to perform a complete surgical excision of the AVF with successful outcome.

The histopathology of traumatic AVF typically reveals dilated thick irregular vessels. The arterial component is demonstrated by visualising the internal elastic lamina, for which elastic stain is required [15]. A traumatic AVF may be indistinguishable from a congenital arteriovenous fistula at the microscopic level [16].

## Conclusions

Traumatic AVF should be considered in the differential diagnosis of a growing pulsatile scalp mass. The diagnosis can be established using angiography (CTA, MRA or DSA). Endovascular treatment is the preferred treatment modality which may be combined with subsequent surgical excision in larger, complex lesions.
